# Homogeneous Age-hardening of Large-sized Al-Sc Foams via Micro-alloying with Zr and Ti

**DOI:** 10.3390/ma17061269

**Published:** 2024-03-09

**Authors:** Xuming Chu, Tianze Wang, Donghui Yang, Xiangyang Peng, Shuo Hou, Shuai Chen, Guangyao Lu, Meiyuan Jiao, Yuan Wu, Andrey A. Rempel, Wentao Qu, Hongxiang Li, Hui Wang

**Affiliations:** 1 State Key Laboratory for Advanced Metals and Materials, University of Science and Technology Beijing, Beijing 100083, China; 2 Beijing Hengxing Yikang Technology Co., Ltd., Beijing 100191, China; 3 College of Mechanics and Materials, Hohai University, Nanjing 210098, China; 4 Equipment Research Center, China Nuclear Powder Technology Research Institute Co., Ltd., Shenzhen 518000, China; 5 Institute of Metallurgy of the Ural Branch of the Russian Academy of Sciences, 620016 Ekaterinburg, Russia; 6 School of Mechanical Engineering, Xi’an Shiyou University, Xi’an 710065, China

**Keywords:** Al-Sc foams, precipitates, age-hardening, micro-alloying, large size

## Abstract

Al-based foams have drawn increasing attention from industry due to their integration of structure and functional properties. However, large-sized Al-based foams still cannot be homogeneously strengthened by long-time aging due to their low thermal conductivity. In this study, we proposed an age-hardening approach that was applied in large-sized Al-0.16Sc-0.17Zr (wt.%) foams via micro-alloying with Zr and Ti compared with Al-0.21Sc foams; it not only achieved homogeneous strength by long-term aging but also reduced the cost of the alloy by substituting Zr and Ti for the more expensive Sc content. The results show that the Al_3_(Sc, Zr, Ti) phase with a core–shell structure as a crucial precipitation strengthening phase by micro-alloying with Zr and Ti was less prone to coarsening after a prolonged aging heat treatment. Therefore, the yielding strength of Al-Sc foam micro-alloying with Zr and Ti remained almost unchanged after a maximum aging time of 1440 h due to less coarsening precipitate, which is consistent with the results of mechanical experiments. These findings provide a new way for the heat treatment strengthening of large-sized Al-based foams, thus promoting their industrial applications.

## 1. Introduction

As a class of ultra-lightweight materials, Al-based foams consisting of solid Al and containing a large volume fraction of gas-filled sealed pores have great potential to be utilized in industrial fields, such as automobile, aerospace, shipment, railway and civil construction, due to their unusual properties [1,2,3,4,5], i.e., good energy absorption capacity; excellent damping insulation of vibration, sound and heat; and low density. Many approaches can be employed to manufacture Al-based foams. Among which, only two methods, namely, melt foaming and powder metallurgy, are industrialized as cost-effective. Nonetheless, the relative low compressive strength of the Al-based foams fabricated by the above two methods, which is usually less than 15 MPa, impedes widespread applications [6,7].

Generally, the mechanical performance of Al-based foams can be improved through either optimizing pore-structures or strengthening the skeleton metal [8,9,10,11]. For instance, for a predetermined porosity, the compressive yielding strength of Al-based foam is increased by either decreasing the porosity, which may have an impact on the lightweight characteristic, or using a strong Al alloy as a starting metal. Therefore, it is the case that alloying followed by an appropriate heat treatment is one of competitive ways to enhance the mechanical properties of Al-based foams [5,12,13,14]. Previous results showed that additions of a small amount of Sc can lead to remarkable improvements in the mechanical properties [15,16,17,18], and such improvements are normally attributed to the precipitation hardening of the Al_3_Sc phase formed during aging after solution heat treatments [19]. It was found that Sc additions have a major impact on strengthening, as they form a fine distribution of spherical L1_2_ dispersoids. However, these effects on the mechanical performance of Al-Sc foams have not been investigated in detail yet. In particular, this improvement is still difficult to achieve in precipitation-strengthened Al-Sc foams with a large size due to their low thermal conductivity. In addition, considering the cost of the additional Sc element, it remains a challenge to obtain high strength from a thermal treatment of large-sized Al-Sc foams with less Sc contained.

There has been extensive work on the addition of Zr together with Sc to allow for the formation of a core–shell L1_2_ structure with a core rich in Sc and a shell rich in Zr [20]. As a result, the addition of Zr together with Sc improves the effectiveness of Sc in Al alloys as an inhibitor of recrystallization and increases the stability of the alloy during prolonged annealing at high temperatures. Therefore, the addition of Zr not only reduces the susceptibility of the Al_3_Sc precipitates to coarsening, finally leading to the high thermal stability of the precipitate, but also reduces the cost of the alloy by substituting Zr and Ti for the more expensive Sc content [21].

In this study, the fabrication of cellular Al-Sc foam samples via micro-alloying with a portion of Zr and Ti instead of Sc was attempted, and then heat treatments of these foams were conducted. The ultra-long-time aging hardening mechanism required for large-sized Al-Sc foams was explored experimentally as well.

## 2. Materials and Methods

A melt-foaming method initially based on the ALPORAS^©^ route was applied to fabricate the cellular Al-0.16Sc-0.17Zr (wt.%) foam samples (Al alloy containing 0.16 wt.% Sc and 0.17 wt.% Zr) with a porosity of ~78% [22]. The specific preparation procedure was elaborated in detail previously [23,24], which can be summarized as follows. A predetermined quantity of Al-0.16Sc-0.17Zr alloy (~1 kg) was melted in a crucible at a fixed temperature and then 2.0 wt.% Ca was introduced into the molten alloy to increase the viscosity of the melt. Then, the thickened Al-Sc melt was foamed by adding a blowing agent, namely, TiH_2_ powder (2.0 wt.%), at a predetermined foaming temperature (T). During the fabrication process, a stirring device was placed inside the melt to ensure a homogeneous distribution of the thickening agent and the blowing agent, and the final pore structures of the products were controlled by adjusting the foaming temperature in the range of 680–710 °C. Finally, the crucible was removed from the furnace, and immediately following, the foamed melt was entirely put into a water tank to solidify it; then, the Al-Sc foam samples were obtained [25]. Meanwhile, cellular Al-0.21Sc foams without Zr micro-addition and pure Al foams were also fabricated by the same route for comparison. The cell wall compositions of the final foams were determined by using an inductive coupled plasma–atomic emission spectrometer (ICP-AES, Pekin-Elmer, Waltham, MA, USA). The porosity (*Pr*) of the foam samples was calculated according to the following equation [26]:

*Pr*(%) = [*V* − (*M*/*ρ_s_*)]/*V* × 100% (1)

where *ρ_s_* is the density of the matrix (2.72 g cm^−3^), and *M* and *V* are the mass and volume of the specimen, respectively.

**Figure 1:**
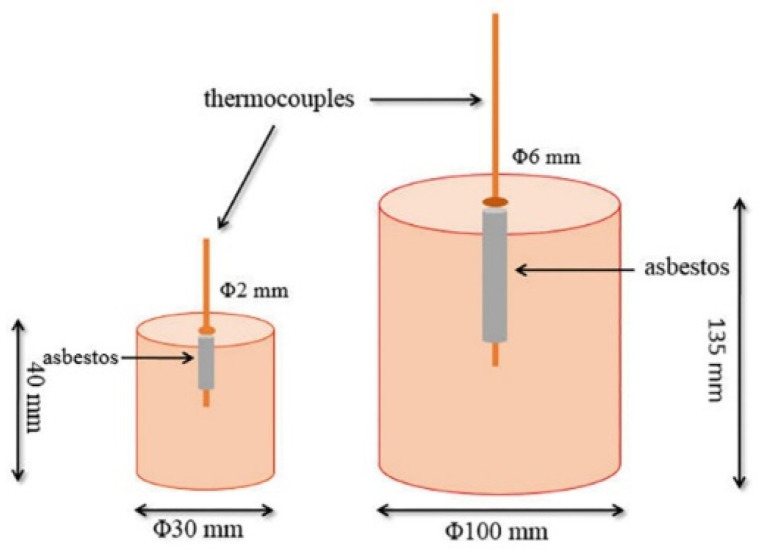
Schematic diagram of core temperature tests for Al-Sc foam samples with two sizes.

According to the previous research results [27,28,29], the thermal conductivities of the Al-based foams are about 2 Wm^−1^ K^−1^, which are much lower than that of the Al matrix. Consequently, the core temperature of a large-sized Al-based foam bulk does not reach the ambient temperature as quickly as that of Al matrix while it is heated, resulting in heterogeneous heating of the metallic framework. In these cases, two Inconel-encased Ni/CrNi thermocouples in this case were inserted into the center of the samples, which were Al-Sc foams with sizes of Φ 30 × 40 mm and Φ 100 × 135 mm, as presented in Figure 1. The temperatures of two thermocouples in the cores, which reflected the core temperature changes during the heating process, were recorded.

The microstructures of the cell walls of the foams were characterized by using an optical microscope (OM, ProgRes C5, Carl Zeiss SMT AG, Oberkochen, Germany), a scanning electronic microscope (SEM, SUPRA 55, Carl Zeiss SMT AG, Oberkochen, Germany) and a high-resolution transmission electronic microscope (HR-TEM, JEM 2100, JEOL Ltd., Tokyo, Japan).

The pore size of the fabricated foam was calculated by analyzing its scanned cross-sectional image with the aid of image analysis software. Briefly, the cross-sectional images of the foam were scanned by a scanner first. Since the intensity of reflected light from the cell edge is stronger than that from the inner surface of the pore, the obtained images can be easily analyzed using Image Pro Plus 3.0 software to acquire the plane pore areas. The equivalent diameter of a plane pore and the equivalent mean pore size can be subsequently calculated.

Specimens in dimension of Φ 24 × 35 mm for compressive tests were cut from the Al-based alloy foam with dimensions of Φ 30 × 40 by an electro-discharging machine, treated by isothermal aging heat treatments with a heating rate of 5 °C min^−1^ and kept from 200 to 600 °C for a period from 2 h to 1440 h (60 days). Some specimens with the same dimensions were also processed by isochronal aging from 300 to 500 °C for comparison. The quasi-static compressive experiments were carried out on the CMT 4350 Universal Testing Machine (Jinan Liangong Testing Technology Co., Ltd., Jinan, China) with a constant strain rate of 3 × 10^−2^ s^−1^ at a room temperature.

## 3. Results and Discussion

### 3.1. Pore Structure and Cell Wall Characterization of Fabricated Al-Sc Foams

**Figure 2:**
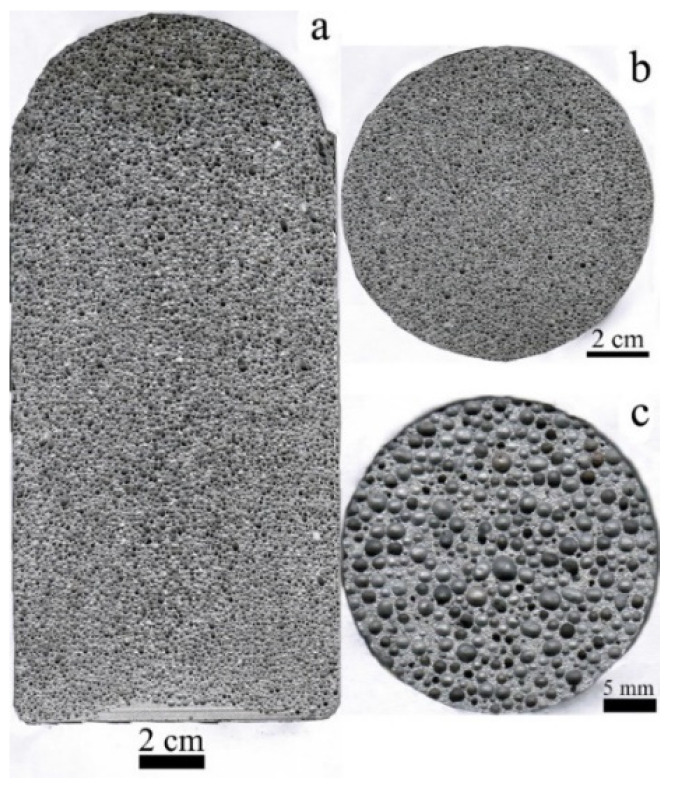
Section images of the cellular Al-0.16Sc-0.17Zr foam with the mean pore size of 1.5 mm: (**a**) longitudinal section image; (**b**) cross-sectional image; (**c**) cross-sectional image of the compressive sample with *Pr* = ~78%.

**Figure 3:**
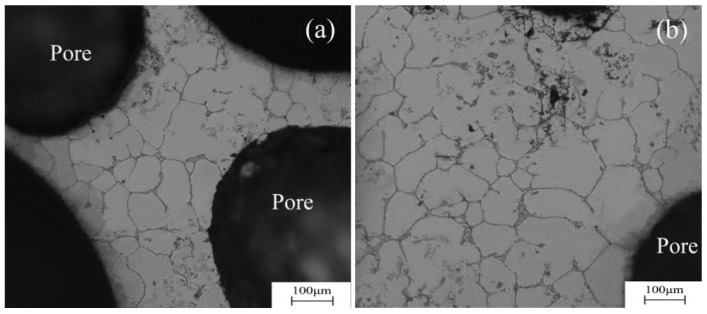
Microscopic structures of the cell walls: (**a**) Al-0.16Sc-0.17Zr foams; (**b**) Al-0.21Sc foams.

As mentioned above, the mechanical properties of Al-based foams are influenced by the following factors: the pore structure, alloying additions and heat treatment. In these cases, the longitudinal sectional image of the foam sample is displayed in Figure 2a, which shows that it has a homogeneous pore structure distribution. The cross-sectional images of the Al-0.16Sc-0.17Zr foams with a porosity of ~78% are displayed in Figure 2b,c.Both the pore structure and porosity are distributed uniformly, indicating successful fabrication. Furthermore, for the Al-Sc alloys, the maximum solid solubility of Sc in Al was 0.38 wt.%, which occurred at the eutectic temperature, which is only 1 °C below the melting point of pure Al, and this low value is increased in practice to over 0.6 wt.% Sc due to non-equilibrium solidification conditions. When the concentration of Sc exceeds the solid solubility, the Al_3_Sc primary phase forms during solidification and such a formation of this primary Al_3_Sc phase can refine the grain size. As shown in Figure 3, the optical microscope images verified the grain sizes of cell walls of the foams, where Al-0.16Sc-0.17Zr and Al-0.21Sc were both approximately 80 μm, implying that the addition of 0.17%Zr had no obvious effect on the grain refinement compared with the Al-0.21Sc alloy without Zr addition.

### 3.2. Inhomogeneity of Aging Temperature and Mechanical Properties of Al-Sc Foams

**Figure 4:**
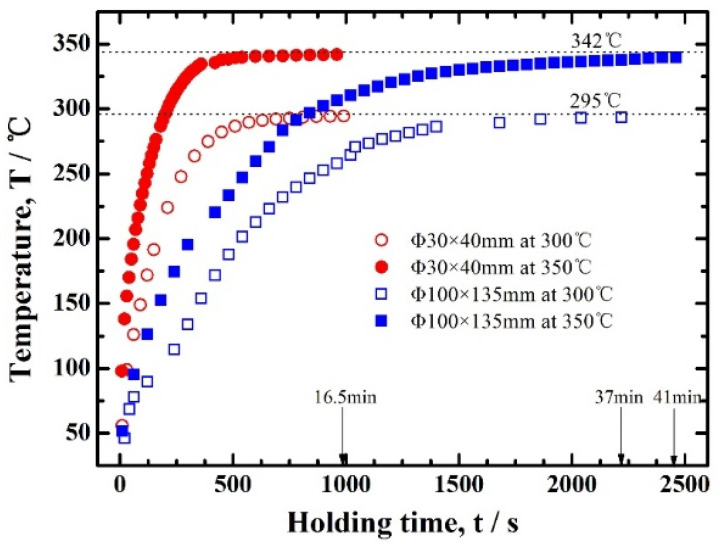
Core temperatures of the Al-0.16Sc-0.17Zr foams with different sizes during the heating at 300 and 350 °C.

The temperature increases of the same Al-0.16Sc-0.17Zr foam samples with different sizes under the same heating conditions exhibited obvious differences in heat treatments, which are presented in Figure 4. The samples with the dimensions of Φ 100 × 135 mm and Φ 30 × 40 mm needed significantly different heating times to reach the temperature of 300 °C. Specifically, the Al alloy foams with the large size was heated to 300 °C after being heated for about 37 min from room temperature. In comparison, the sample with the small size only needed 16.5 min to reach the same temperature.

In these cases, which is known as Newtonian heating, the surface-area-to-volume ratio of the sample had a significant influence on the inhomogeneity in the temperature distribution. The temperature *T* in the sample can be expressed by

*T*(*t*) = *T_end_* − (*T_end_* − *T_start_*) *exp*(−*t*/*t_au_*) (2)

where *t_au_* is proportional to the mass-to-surface ratio or, for a given porosity, to the volume-(V)-to-surface-(A) ratio. Herein, the proportionality constant is in essence the ratio of the heat transfer coefficient at the surface and the volumetric heat capacity, which, for a predetermined porosity (~78% in this case), is a constant. The surface-(A)-to-volume-(V) ratios (A/V) of the two samples with the dimensions of Φ 100 × 135 mm and Φ 30 × 40 mm were 0.55 cm^−1^ and 1.83 cm^−1^, respectively, demonstrating that the heating of the small sample was about 3.3 times faster than that of the larger one. The results agreed with the different heating rates of the two samples in Figure 4, in which the heating rate of the small sample was 2.24 times faster than that of the large sample, considering other heating transferring effects. It can be concluded that the heating surface-area-to-volume ratio played an important role in the homogeneous temperature distribution rather than low thermal conductivity.

In terms of an industrial large-sized Al foam bulk product, it usually has a dimensional size of 2.5 m × 1.5 m × 0.6 m, for which A/V was calculated as 0.055 cm^−1^, which is much smaller than that of the above two samples. Therefore, homogeneous aging heat treatment needs a long time due to the low thermal conductivity rather than the A/V ratio.

**Figure 5:**
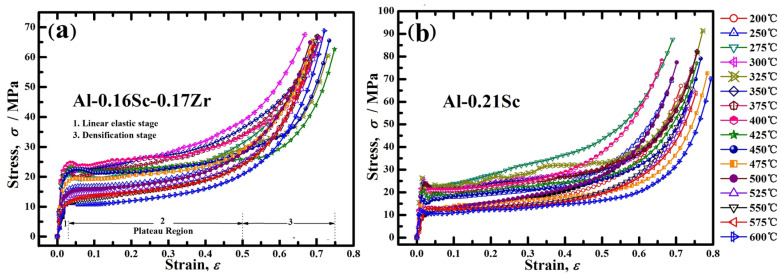
Stress–strain curves of the foams in different aging stage: (**a**) Al-0.16Sc-0.17Zr; (**b**) Al-0.21Sc.

To determine the appropriate peak aging temperature, the aging treatments on the two Al-Sc foams, namely, Al-0.16Sc-0.17Zr and Al-0.21Sc, in various temperatures were processed; then, quasi-static compression tests were conducted, where each test was repeated three times and the average of the numerical values was taken to ensure the accuracy. Compressive stress–strain curves of Al alloy foams, namely, the Al-0.16Sc-0.17Zr foams and Al-0.21Sc foams, with the same porosity of ~78% are shown in Figure 5a,b, respectively. These foams typically display three regions [30]: an initial, approximately linear deformation region until the peak stress; followed by a plateau region, where the stress was almost a constant; and the final densification region in which the stress increased steeply due to the fact that collapsed cells were almost fully compacted together. Herein, all 17 samples with different aging temperatures from 200 to 600 °C with steps of 25 °C were tested in Figure 5. The ends of the plateau range and final densification range were selected as the strains at 50% and 75%, respectively.

### 3.3. Effect of Homogeneous Precipitates Distribution on the Yielding Strength of Al-Sc Foams Micro-Alloyed with Zr and Ti

**Figure 6:**
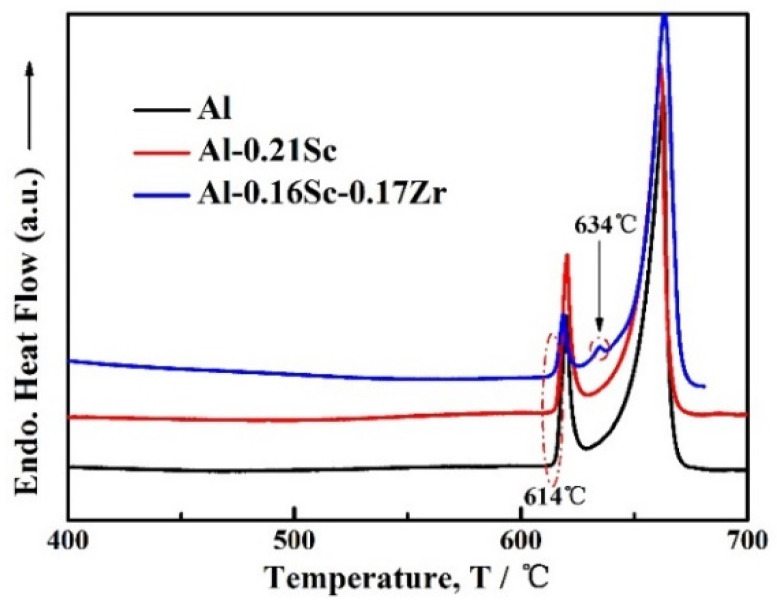
DSC curves of as-cast Al-0.16Sc-0.17Zr foams, as-cast Al-0.21Sc foams and Al foams.

An Al matrix can be precipitation-strengthened via micro-alloying with Sc, where the strength is improved by heat treatment aging [12]. Many studies have identified some substitutional elements, i.e., Zr and Ti, for Sc in the Al–Sc-based alloys to decrease the use of the expensive Sc, increase the ambient strength and increase the service temperature while maintaining a high strength [31,32,33]. Therefore, in general, the micro-addition of Zr is a highly attractive strategy in lowering the cost. In order to appropriately determine the heat treatment parameters for the Al-Sc-Zr/Al-Sc foams, differential scanning calorimetry (DSC, FDSC-800B, Shanghai Yanjin Scientific Instruments Co., Ltd., Shanghai, China) experiments for three samples, namely, as-cast Al-0.16Sc-0.17Zr, Al-0.21Sc and pure Al foams, were carried out in an Ar gas flow with 40 mL min^−1^ and at a heating rate of 5 °C min^−1^. According to the Al-Ca phase diagram [34], α-Al and Al_4_Ca undergo a eutectic reaction at 616 °C during the solidification process. As shown in Figure 6, for all Al-based foams, there was an endothermic peak starting at the temperature of 614 °C, which was most probably caused by the phase transformation of an intermetallic compound Al_4_Ca, where the Ca was from dissolving the thickening agent and the transformation temperature of the Al_4_Ca phase was approximately 616 °C. Furthermore, there was an additional endothermic peak that appeared around 634 °C for the Al-0.16Sc-0.17Zr foams [35], which was most probably caused by the phase transformation of primary Al_3_(Sc, Zr, Ti) based on the research results of the Al-Sc-Zr-Ti alloys, although the phase transitions are not yet fully clear [36,37], where Ti was generated by the decomposition of the blowing agent, namely, TiH_2_. With the temperature increase, all the alloy was melted, corresponding to the endothermic peaks with peak temperature of 660 °C due to the eutectic reaction *L* → *α-Al* + *Al_3_Sc*. In this case, the solution treatment temperature should, therefore, be chosen to be below 614 °C in order to avoid over-burning because of the presence of Al_4_Ca.

**Figure 7:**
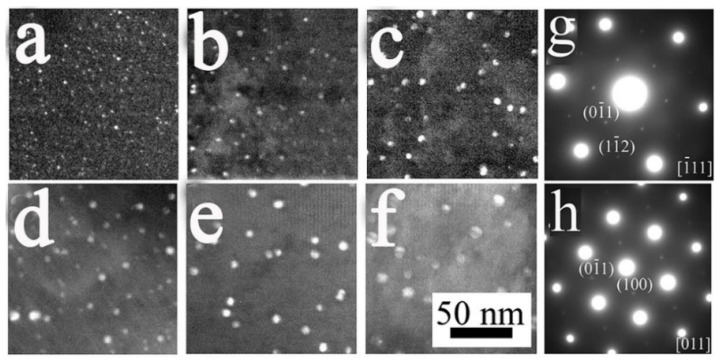
Comparison of precipitates, as observed by employing TEM images (utilizing the $0\bar{1}1$ superlattice reflection in the $\left[1\bar{1}1\right]$ and [011] zone axes) of the Al-0.16Sc-0.17Zr (**a**–**c**) and Al-0.21Sc foams (**d**–**f**) after isothermal aging at 400 °C for 3 h, 24 h and 120 h, respectively; (**g**,**h**) are selected area electron diffraction images for Al-0.16Sc-0.17Zr foams and Al-0.21Sc foams, respectively. The magnification scale for the figures is the same as in (**f**).

TEM investigations for the precipitates in the cell walls of the Al-0.16Sc-0.17Zr foams after the isothermal aging at 400 °C for 3 h, 24 h and 120 h were conducted, as presented in Figure 7, and the Al-0.21Sc foams without Zr micro-alloying were processed similarly for comparison. As shown in Figure 7a–c, which were taken along the $\left[1\bar{1}1\right]$ zone axis, and in the selected area diffraction pattern (Figure 7g), the dark-field images of the Al-0.16Sc-0.17Zr foams indicate a clearly homogeneous distribution of the Al_3_(Sc, Zr, Ti) precipitates, and such precipitates constantly kept a small size after the corresponding aging times for 3 h, 24 h and 120 h. In comparison, the dark-field images of the Al-0.21Sc foams had no Zr micro-alloying, as indicated in Figure 7d–f, which were taken from the [011] zone axis (Figure 7h); this demonstrates that both precipitates were a little bit coarsened after aging times of 3 h, 24 h and 120 h.

The multi-faceted nature of Al_3_Sc precipitates was reported in great detail [38,39], and Al_3_(Sc, Zr) precipitates with a cuboid core–shell structure in Al-Sc alloys with the micro-addition of Zr were also observed. During the aging of the Al-Sc micro-alloying with Zr, the Zr was pushed to the boundaries of the precipitate as the Al_3_Sc precipitate continued to grow. The content of Zr around the precipitate was also enriched at a significant composition gradient, along with the diffusion of Zr in the matrix. The Zr in the precipitate and the matrix aggregated at the edge of the precipitate with the growth of the precipitate. This could form a stable core–shell structure with the Al_3_Sc precipitate as the core and the Al_3_(Sc, Zr) precipitate as the shell. The presence of these core–shell cuboids can be counter-intuitive, as one would expect that the segregation of Zr would decrease the interfacial free energy, which would lead to the stabilization of the spheroidal particles. In fact, the core–shell structure was driven by the significant difference in diffusivities between the Sc and Zr. The use of multi-step aging treatments was found to be useful in separating the formation of the core at lower temperature and the shell at higher temperature to form a distribution of small and thermally stable precipitates. As shown in Figure 7, the precipitates at different aging times indicated that this core–shell structure of Al_3_(Sc, Zr, Ti) was more stable than that of Al_3_Sc. The growth rate of the Al-Sc-Zr alloy was reduced to have better thermal stability performance.

**Figure 8:**
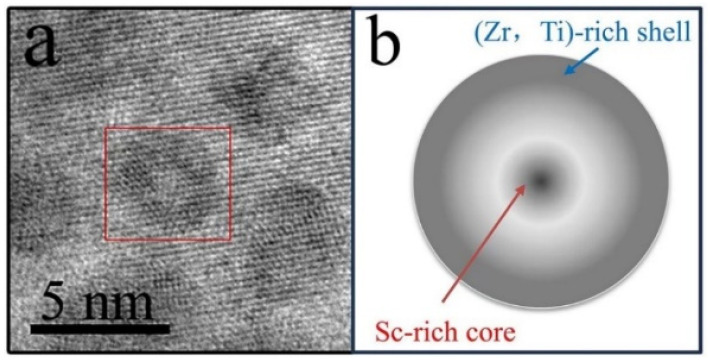
(**a**) HR-TEM image of Al_3_(Sc, Zr, Ti) precipitates in Al-0.16Sc-0.17Zr after aging at 500 °C; (**b**) the supposed schematic diagram of Al_3_(Sc, Zr, Ti) precipitate.

Previous studies verified that Zr and Ti can also substitute for Sc in L1_2_-ordered Al_3_(Sc, Zr, Ti) precipitates, and the chemical composition of Al_3_Sc precipitates is anticipated to change with Zr additions due to Zr enrichment near the precipitate/matrix hetero-phase interface, which could alter the precipitate/dislocation interaction [40,41]. The concentration of the slow-diffuse Zr is sufficient to provide coarsening resistance at 400 °C for up to 66 days by forming a Zr-enriched outer shell that encapsulates the precipitates [42]. In this case, it can therefore be inferred that Al_3_(Sc, Zr) precipitates exhibited significantly higher coarsening resistance compared with Al_3_Sc precipitates in the Al–Sc binary alloys owing to the sluggish diffusivity of Zr in α-Al [41]. A Zr-enriched precipitate shell was formed during the aging and served as a diffusion barrier for Sc, which was enriched in the precipitate. For microalloying with Ti, it could reduce the lattice parameter mismatch between the α-Al and Al_3_Zr precipitates, which led to the corresponding interfacial energy decrease [33]. The diffusivity of Ti was lower than that of Zr and Sc, and therefore, it can be inferred that Ti could only replace some Zr in the outer shell of precipitates at a relative high aging temperature. In this case, as shown in Figure 8a, the core–shell structure precipitate, which is indicated by the red square, was observed in the cell wall of the Al-0.16Sc-0.17Zr foams after isochronal aging at 500 °C. Subsequently, it was supposed that the ordered-L1_2_ precipitate formed such a core–shell structure, as shown in the schematic diagram in Figure 8b, was composed of a core of Al_3_Sc enriched Sc, surrounded by a shell of Al_3_(Sc, Zr, Ti) that was slightly enriched in Zr and Ti. The nanosized precipitation composition will be further characterized as a follow-up study.

**Figure 9:**
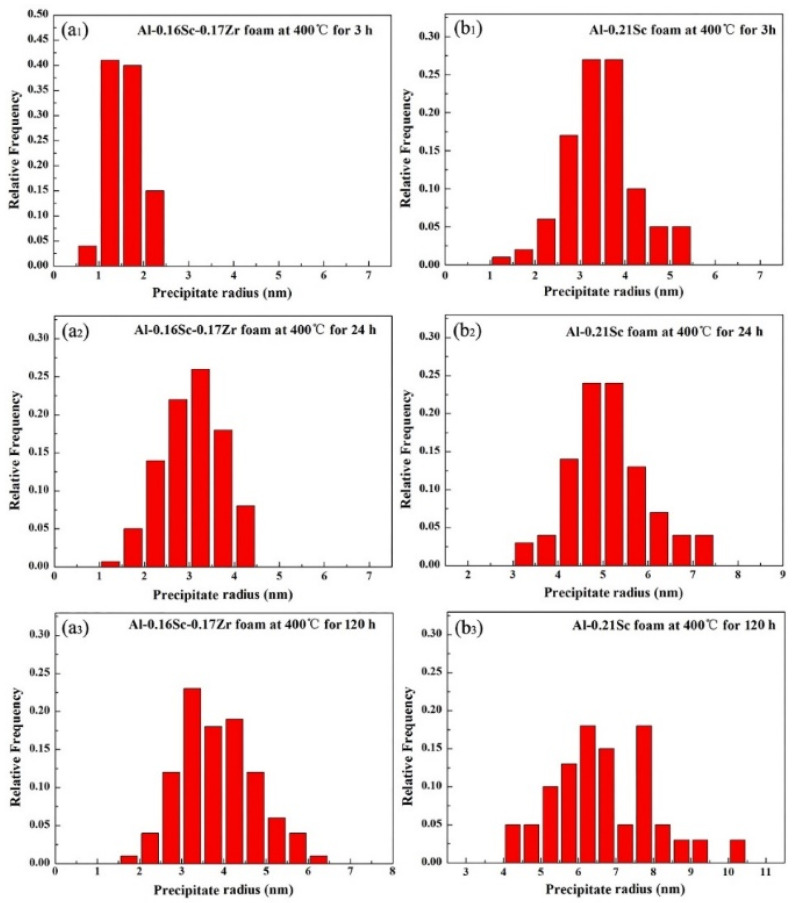
The size distributions of precipitates in (**a1**–**a3**) the Al-0.16Sc-0.17Zr foam and (**b1**–**b3**) the Al-0.21Sc foam after direct isothermal aging at 400 °C for 3 h, 24 h and 120 h.

The size distribution of precipitates in the Al-0.16Sc-0.17Zr foam and Al-0.21Sc foam for comparison after direct isothermal aging at 400 °C for 3 h, 24 h and 120 h was measured to evaluate the coarsening of precipitates in Figure 9. Specifically, as a result, the precipitate radius of the Al-0.16Sc-0.17Zr foam at 400 °C for 3 h were distributed in the range of 0.5 to 2.5 nm, as displayed in Figure 9(a1), which, in comparison, was much smaller than that of the Al-0.21Sc foam in the range of 1.0 nm to 5.5 nm after the same heat treatment process, as displayed in Figure 9(b1). Moreover, the distributions of the precipitate radius in the Al-0.16Sc-0.17Zr foam for 24 h and 120 h, as shown in Figure 9(a2,a3), were 1.0 nm to 4.5 nm and 1.5 nm to 6.5 nm, respectively. In contrast, the distributions of the precipitate radius in the Al-0.21Sc foam for 24 h and 120 h, as displayed in Figure 9(b2,b3), were 3.0 nm to 7.5 nm and 4.0 nm to 10.5 nm, respectively. All the results also show that the Al_3_(Sc, Zr, Ti) precipitates with this core–shell structure did not grow much after the prolonged aging and the micro-alloying with Zr and Ti compared with the Al-Sc foam with more Sc contents without Zr addition effectively restrained the kinetics of the precipitation coarsening.

**Figure 10:**
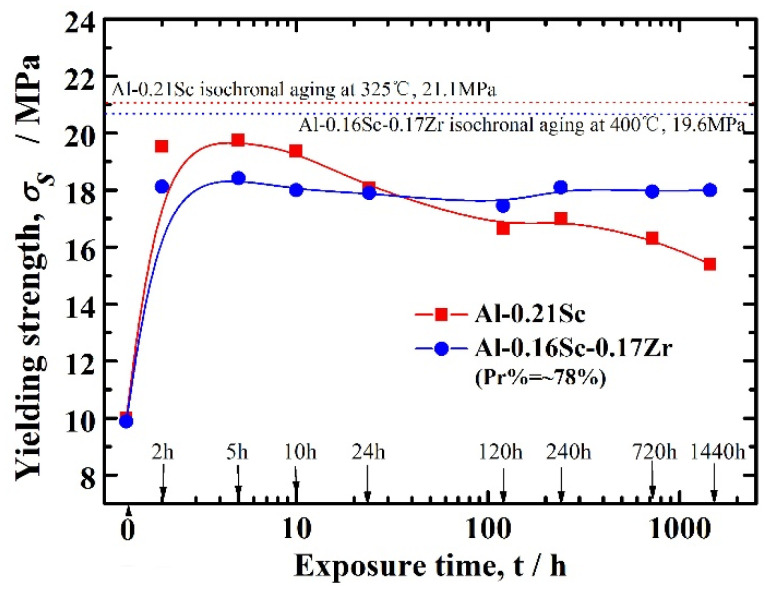
The yielding strengths of Al-Sc foams after different isothermal aging times at 300 °C.

The yielding strengths of two Al-Sc foams after different time spent aging at 300 °C are displayed in Figure 10, which also verified the effects of precipitation strengthening with long-term homogeneous aging on the mechanical properties. As shown in Figure 10, peak yield strengths of two foams, namely, Al-0.16Sc-0.17Zr foams and Al-0.21Sc foams, were achieved after 5 h of isothermal aging treatment. After the long-time aging processing, the yield strength of the Al-0.16Sc-0.17Zr foams remained almost consistent as the aging time increased, even after 1440 h, due the homogeneous precipitation strengthening effect. Meanwhile, the yielding strength of the Al-0.21Sc foams kept steadily decreasing compared with that of the Al-0.16Sc-0.17Zr foams, although the peak yielding strength value of the Al-0.21Sc foam (19.75 MPa) was higher than that of the Al-0.16Sc-0.17Zr foams (18.42 MPa) in the initial 24 h isothermal aging treatment. Notably, after the initial 24 h isothermal aging treatment, the yielding strength of the Al-0.21Sc foams was lower than that of the Al-0.16Sc-0.17Zr foams, while the yielding strength of the Al-0.16Sc-0.17Zr foams always maintained a high strength level as the aging time increased and finally surpassed the decreased strength of the Al-0.21Sc foams. The results also confirmed that Zr and Ti as substitutional elements for Sc in Al-Sc based alloys not only decreased the use of the expensive Sc, but also increased the ambient yielding strength of Al-Sc foams.

Therefore, it was concluded that the yielding strength of Al-0.16Sc-0.17Zr foams could maintain unchanged level after a long time of aging, which is of special significance for large-sized Al-based foams to obtain homogeneous strength. In other words, the homogeneous precipitation of large-sized Al-Sc alloy foams was obtained via long-time aging heating, then these large-sized Al-Sc alloy foams were uniformly strengthened, which is more attractive for practical applications. In this case, the unchanged yielding strength of Al-0.16Sc-0.17Zr foams undergoing the isothermal aging at 300 °C within the period of 1440 h suggests that the large-sized Al alloy foams with micro-additions of Zr and Ti needed more aging time to be strengthened by the homogeneous precipitation heat treatment. In addition, the peak yielding strengths of Al-0.16Sc-0.17Sc foams and Al-0.21Sc foams after isochronal aging were 19.6 MPa and 21.1 MPa, respectively.

## 4. Conclusions

In this work, the effects of aging treatments on the microstructures and mechanical property of Al-0.16Sc-0.17Zr foams and Al-0.21Sc alloy foams with a porosity of ~78%, which were fabricated by a melt-foaming method, were investigated and we proposed an age-hardening approach, which was applied in large-sized Al-Sc alloy foams via micro-alloying with Zr and Ti. Owing to the Al_3_(Sc, Zr, Ti) phase with a core–shell structure that was less prone to coarsening of homogeneous precipitates after prolonged aging, the yielding strength of Al-Sc foams micro-alloyed with Zr and Ti remained almost stable after a maximum of 1440 aging hours, which is of great significance for the heat treatment strengthening of large-sized Al-based foams. Our findings provide a new method for the heat treatment strengthening of large-sized Al-based foams, thus promoting their industrial applications.

## Data Availability

Data are contained within the article.
